# Effect of abiraterone acetate plus prednisone on the QT interval in patients with metastatic castration-resistant prostate cancer

**DOI:** 10.1007/s00280-012-1916-9

**Published:** 2012-07-03

**Authors:** A. W. Tolcher, K. N. Chi, N. D. Shore, R. Pili, A. Molina, M. Acharya, T. Kheoh, J. J. Jiao, M. Gonzalez, A. Trinh, C. Pankras, N. Tran

**Affiliations:** 1 South Texas Accelerated Research Therapeutics (START), Center for Cancer Care, 4383 Medical Drive, San Antonio, TX 78229 USA; 2 British Columbia Cancer Agency, Vancouver, BC USA; 3 Carolina Urologic Research Center, Myrtle Beach, SC USA; 4 Roswell Park Cancer Institute, Buffalo, NY USA; 5 Janssen Research & Development LLC, Raritan, NJ USA

**Keywords:** Abiraterone acetate, Pharmacokinetics, QT interval, Castration-resistant prostate cancer, Phase 1

## Abstract

**Purpose:**

Abiraterone is the active metabolite of the pro-drug abiraterone acetate (AA) and a selective inhibitor of CYP17, a key enzyme in testosterone synthesis, and improves overall survival in postdocetaxel metastatic castration-resistant prostate cancer (mCRPC). This open-label, single-arm phase 1b study was conducted to assess the effect of AA and abiraterone on the QT interval.

**Methods:**

The study was conducted in 33 patients with mCRPC. Patients received AA 1,000 mg orally once daily + prednisone 5 mg orally twice daily. Electrocardiograms (ECGs) were collected in triplicate using 12-lead Holter monitoring. Baseline ECGs were obtained on Cycle 1 Day-1. Serial ECG recordings and time-matched pharmacokinetic (PK) blood samples were collected over 24 h on Cycle 1 Day 1 and Cycle 2 Day 1. Serial PK blood samples were also collected over 24 h on Cycle 1 Day 8.

**Results:**

After AA administration, the upper bound of the 2-sided 90 % confidence interval (CI) for the mean baseline-adjusted QTcF change was <10 ms; no patients discontinued due to QTc prolongation or adverse events. No apparent relationship between change in QTcF and abiraterone plasma concentrations was observed [estimated slope (90 % CI): 0.0031 (−0.0040, 0.0102)].

**Conclusions:**

There is no significant effect of AA plus prednisone on the QT/QTc interval in patients with mCRPC.

## Introduction

Androgen receptor activation and signaling by androgens originating from other sources or *de novo* synthesis play an important role in the progress of castration-resistant prostate cancer (CRPC) [1, 2]. Inhibiting the systemic biosynthesis of androgens by targeting 17α-hydroxylase/C17,20-lyase (CYP17), an enzyme that catalyzes two key steroid reactions involving CYP17 in the androgen biosynthesis pathway, represents a rational therapeutic approach in the treatment of CRPC [1].

Abiraterone is a selective, potent, and irreversible inhibitor of CYP17, with an IC50 of 2–4 nM for the hydroxylase and lyase [3]. Abiraterone acetate (AA; ZYTIGA^**®**^, Janssen Research & Development LLC, United States), the 3-acetate analog of abiraterone is a pro-drug [4]. Abiraterone is the predominant active metabolite of AA detected in plasma both in preclinical [5] and clinical studies [6]. AA (ZYTIGA^**®**^) in combination with prednisone is approved in the United States [7], Canada [8], and Europe [9] for the treatment of metastatic CRPC (mCRPC) in patients who have received prior chemotherapy containing docetaxel.

The primary objective of this phase 1b study was to assess the potential effect of AA plus prednisone on the QT/QTc interval by using pharmacokinetic (PK) and time-matched electrocardiograms (ECGs) in patients with mCRPC. The study also assessed PK and safety of AA. The follow-up period of the study to assess survival is ongoing, and results of the analyses of all ECGs, PK, and safety data through C2D2 are presented here.

## Methods

### Study population

Patients with mCRPC, with progressive disease following gonadotropin-releasing hormone (GnRH) therapy or surgical castration, with no more than 1 course of prior chemotherapy, were enrolled. Patients had confirmed adenocarcinoma of the prostate without neuroendocrine differentiation or small cell histology; metastatic disease documented by bone scan, CT or MRI; prostate-specific antigen (PSA) progression according to Prostate Cancer Working Group 2 (PCWG2) criteria [10] or radiographic progression according to Response Evaluation Criteria in Solid Tumors (RECIST) criteria [11]; testosterone levels <50 ng/dL (<2.0 nM); Eastern Cooperative Oncology Group (ECOG) Performance Status score [12] ≤1; and adequate hematologic and biochemical indices.

Exclusion criteria were serious or uncontrolled coexistent non-malignant diseases; uncontrolled hypertension; clinically significant heart disease in the past 6 months; diagnosis of cardiac arrhythmia with abnormal ECG; malignancies other than non-melanoma skin cancer; surgery or local prostatic intervention, radiotherapy, or immunotherapy within 30 days of the first dose; prior chemotherapy with mitoxantrone or other anthracyclines; previous treatment with AA or other investigational CYP17 inhibitor or investigational antiandrogens.

This study was conducted in accordance with the ethical principles originating in the Declaration of Helsinki and in accordance with ICH Good Clinical Practice guidelines, applicable regulatory requirements, and in compliance with the protocol. Written informed consent was obtained from all patients. The study was approved by the institutional review board of all participating centers and is registered on ClinicalTrials.Gov (NCT00910754).

### Study design

This open-label, single-arm study had a 14-day screening period. The treatment period consisted of 28-day treatment cycles. Patients received AA 1,000 mg (4 × 250 mg tablets) once daily plus prednisone 5 mg tablets twice daily, beginning on C1D1 (there was no C1D0). Treatment continued until disease progression.

Serial sets of 3 time-matched ECG measurements using a 12-lead Holter monitor were obtained over 24 h on C1D-1 (baseline), and at predose and 0.5, 1, 1.5, 2, 3, 4, 6, 8, 12, and 24 h postdose on C1D1and C2D1. Twelve-lead ECGs were interpreted and annotated in random order by a central over-reading board-certified cardiologist who was blinded to time and date of the recording. Blood samples for PK analysis of abiraterone and AA levels were collected from each patient at predose and at 0.25, 0.5, 1, 1.5, 2, 3, 4, 6, 8, 12, and 24 h postdose on C1D1 and C1D8. Samples were also collected on C2D1 at predose and 0.5, 1, 1.5, 2, 3, 4, 6, 8, 12, and 24 h postdose. Samples were time-matched to ECG measurements on C1D1 and C2D1. Predose samples were also collected on C1D6 and C1D7 to confirm the attainment of steady-state abiraterone concentration levels. Samples were analyzed using ultra performance liquid chromatography with tandem mass spectrometric detection. The standard curve range was 0.200–200 ng/mL for abiraterone and was 0.200–50 ng/mL for AA. The follow-up period of the study for collection of survival data is ongoing.

### Study evaluations

#### Pharmacodynamic evaluations

Heart Rate (HR), PR interval, QRS duration, and QT interval [using Bazett’s (QTcB = QT/RR^1/2^) and Fredericia’s correction (QTcF = QT/RR^1/3^), where QT, RR, QTcB, and QTcF are expressed in milliseconds] were measured on each ECG. Each ECG was graded as Normal (NML), Abnormal, Clinically Insignificant (ACI), or Abnormal, Potentially Clinically Significant (APCS).

The primary endpoint was the mean maximal change from baseline in QTcF. Secondary endpoints included pharmacokinetic–pharmacodynamic interactions and proportion of patients with change from baseline QTc > 30 ms or >60 ms at any time after first dose of study drug.

#### Pharmacokinetic evaluations

Maximum plasma concentration (*C*_max_); time to reach *C*_max_ (*t*_max_); area under the plasma concentration–time curve (AUC) from 0 to 24 h (AUC24 h) as calculated by the linear trapezoidal rule for increasing concentrations and the logarithmic trapezoidal rule for decreasing concentrations; accumulation ratio for Cmax (AR Cmax); and accumulation ratio for AUC (AR AUC) were calculated from plasma concentration–time data for abiraterone using non-compartmental analysis with WinNonlin version 5.2.1 (Pharsight Corporation, Mountain View, CA, USA).

### Statistical methods

The mean maximal QTcF change from baseline in patients treated with AA is expected to be approximately 7 ms [standard deviation (SD) of 8.5 ms]. Using a two-sided test at the 5 % level of significance, a sample size of approximately 34 patients provides 90 % power to detect a QTcF change from baseline >5 ms.

For all ECG parameters (HR, QTcF, QTcB, PR, QRS, and QT interval) up to C2D2, the two-sided 95 % confidence intervals (CIs) were provided for the mean ECG parameters and two-sided 90 % CIs were provided for mean change from baseline. Qualitative variables were presented as category counts and percentages. The QTcF was considered prolonged if one of the three criteria occurred: an increase from baseline in QTcF of >30 ms but <60 ms; a ≥60 ms increase; and an increase to >500 ms in QTcF intervals.

Results were analyzed for patients’ response after the initial dose of AA (C1D1) and then again during steady state (C2D1). Three sets of categorical descriptions were performed: summary of the clinical interpretations of the ECGs (NML, ACI, and APCS) for all ECG time points; summary of the changes from baseline to all ECG time points using the following categories for QTcF and QTcB: ≤0 ms; >0 ms but <30 ms; ≥30 ms but <60 ms; or ≥60 ms; and finally any QTc of ≤450 ms; any QTc >450 ms but ≤480 ms; any QTc >480 ms but ≤500 ms; or any QTc >500 ms.

Summary of PK results was evaluated over the same time points as ECG extractions after initial dose and at steady state for correlation between compound concentration and QTcF readings. To explore any pharmacokinetic–pharmacodynamic relationship for abiraterone, in addition to graphical method, a linear mixed-effects model was fit to the data with change from baseline in QTcF as dependent variable and abiraterone concentration as a predictor and subject as a random effect.

### Safety evaluations

Safety evaluations included treatment-emergent adverse events (TEAEs) reported throughout the study, changes in vital sign measurements, clinical laboratory test results, and physical examinations.

## Results

### Baseline demographics and disease characteristics

Thirty-three patients with confirmed diagnosis of mCRPC were enrolled from June 06, 2009 through November 27, 2009 at 4 centers. Patients had a mean (SD) age of 65 (11.5) years and all were white. The baseline disease characteristics are given in Table 1. None of the patients had a left ventricular ejection fraction <50 %.

**Table 1:** Baseline disease characteristics (safety analysis set)

	*N* = 33
Years since diagnosis to first dose	
Mean (SD)	8.6 (6.57)
Median (range)	6.7 (1, 32)
Stage at diagnosis, *n* (%)	
Primary tumor (T) stage	
T1–T4	23 (69.7 %)
TX	7 (21.2 %)
Unknown/not applicable	3 (9.1 %)
Regional lymph nodes (N) Stage, *n* (%)	
N0–N3	11 (33.3 %)
NX	17 (51.5 %)
Unknown/not applicable	5 (15.2 %)
Metastasis (M) stage at diagnosis, *n* (%)	
M0	8 (24.2 %)
M1 (M1, M1a, M1b, M1c)	15 (45.5 %)
MX	8 (24.2 %)
Unknown/not applicable	2 (6.1 %)
Gleason total score at diagnosis, *n* (%)	
<7	3 (9.1 %)
7^a^	16 (48.5 %)
>7	11 (33.3 %)
Unknown	3 (9.1 %)
PSA (ng/mL) at initial diagnosis^b^	
Mean (SD)	213.7 (538.28)
Median (range)	11.7 (2, 2183)
Extent of disease, *n* (%)	
Bone only	14 (42.4 %)
Visceral disease only	1 (3.0 %)
Bone and soft tissue (node only)	8 (24.2 %)
Bone and visceral disease	4 (12.1 %)
Bone, soft tissue (node only), and visceral disease	3 (9.1 %)
Soft tissue only (node only)	1 (3.0 %)
Soft tissue (node only) and visceral disease	2 (6.1 %)
ECOG status, *n* (%)	
0	26 (78.8 %)
1	7 (21.2 %)
MUGA scan/echocardiogram, *n* (%)	
Normal	30 (90.9 %)
Abnormal-NCS	3 (9.1 %)
Prior chemotherapy, *n* (%)	
Yes	9 (27 %)^c^
No	24 (73 %)
Prior radiotherapy, *n* (%)	
Yes	23 (70 %)
No	9 (27 %)
Unknown	1 (3 %)
Prior orchiectomy, *n* (%)	
Yes	5 (15 %)
No	28 (85 %)
Prior therapy with ketoconazole or aminoglutethimide, *n* (%)	
Yes	6 (18 %)
No	27 (82 %)
Number of prior chemotherapies, mean (SD)	1.0 (0.00)

*ECOG* Eastern Cooperative Oncology Group, *MUGA* Multi-gated Acquisition Scan, *NCS* non-clinically significant, *PSA* prostate-specific antigen

^a^One patient with total Gleason grade of 7 and unknown first and second Gleason grade is included in this category

^b^ *n* = 25,

^c^All 9 patients were on docetaxel

### Pharmacodynamic results

#### Analysis of QTcF

The mean QTcF changes remained stable on C1D1 (−6.0 to 2.3 ms) and on C2D2 (−11.9 to −1.7 ms) (Table 2). The upper limit of the 90 % CI of the mean baseline corrected QTcF change at each postdose time point was below 10 ms on C1D1 (maximum of upper limits = 5.4 ms) and C2D1 (maximum of upper limits = 2.4 ms).

**Table 2:** Summary statistics for change from baseline of QTcF interval (pharmacodynamic analysis set)

Cycle	Days	Time point	*N*	Mean (SD) ms	Median ms	Min:Max Ms	Q1:Q3 ms	90 % CI ms
1	1	Predose	31	1.7 (15.5)	−0.3	−30.3:45.7	−8.3:8.0	−3.0:6.4
		0.5	32	2.3 (10.1)	2.7	−25.3:23.0	−5.2:9.5	−0.8:5.3
		1	32	−0.5 (11.4)	−2.0	−18.0:27.7	−9.7:4.8	−4.0:2.9
		1.5	32	0.2 (14.3)	−0.3	−23.7:27.7	−10.7:11.3	−4.1:4.5
		2	32	−1.7 (10.0)	−2.0	−20.0:19.0	−8.5:5.3	−4.6:1.3
		3	31	−2.9 (12.0)	−5.3	−24.3:20.7	−12.7:5.7	−6.5:0.8
		4	31	−3.0 (8.0)	−1.0	−20.3:11.7	−7.3:2.3	−5.4:−0.6
		6	31	1.4 (13.0)	1.0	−35.0:21.7	−7.7:14.7	−2.5:5.4
		8	32	−1.4 (13.1)	−1.3	−44.0:22.7	−9.3:6.2	−5.3:2.5
		12	31	0.3 (11.9)	4.0	−37.7:22.0	−7.0:6.7	−3.4:3.9
		24	28	−6.0 (9.7)	−5.2	−37.0:6.7	−8.5:−1.2	−9.1:−2.9
2	1	Predose	29	−3.4 (13.2)	−3.3	−38.7:18.3	−13.3:6.7	−7.6:0.8
		0.5	32	−3.1 (11.8)	−3.2	−32.0:18.3	−9.7:5.7	−6.6:0.5
		1	33	−1.7 (13.9)	0.0	−31.7:20.3	−11.3:9.0	−5.8:2.4
		1.5	33	−4.2 (15.6)	−1.0	−37.7:34.0	−13.7:4.0	−8.8:0.4
		2	33	−4.7 (12.1)	−3.3	−27.0:19.7	−14.0:4.0	−8.3:−1.1
		3	33	−5.0 (15.4)	−2.0	−42.0:19.0	−15.7:7.3	−9.6:−0.5
		4	32	−6.2 (14.4)	−5.3	−30.3:28.7	−17.7:3.3	−10.5:−1.9
		6	30	−3.1 (17.6)	−6.2	−42.0:27.0	−12.0:9.3	−8.6:2.4
		8	32	−3.1 (16.0)	−6.2	−37.0:35.7	−12.5:7.7	−7.9:1.7
		12	33	−2.9 (11.2)	−4.7	−21.7:23.3	−9.0:4.3	−6.2:0.4
		24	29	−11.9 (14.2)	−12.7	−38.0:21.7	−20.7:−3.7	−16.4:−7.4

Two patients with normal predose value had a postdose QTcF value >450 ms. The number of patients with at least one QTcF value >450 ms but ≤480 ms were 11 on C1D-1 (baseline), 9 on C1D1, and 7 on C2D1. No patient had a QTcF > 480 or >500 ms or a change in QTcF > 60 ms at any time point. One patient had a C2D1 predose QTcF value of 480 ms (an increase of 8.7 ms from time-matched baseline values). Two patients each had QTcF changes ≥30 ms but <60 ms at C1D1 predose and on C2D1.

#### Heart rate, PR interval, QRS duration, and changes in ECG status

Heart rate was stable on C1D1and C2D1, with all mean postdose HR changes being less than or equal to 5 beats per minute (bpm). The categorical analysis was based on a normal HR between 60 and 100 bpm (Common Terminology Criteria for Adverse Events [CTCAE] Version 4.0 guidelines). Nineteen patients had HR below 60 bpm at one or more time points; of these, 10 had normal baseline values. Nine patients had HR above 100 bpm at one or more time points; of these, 7 had normal baseline values. These changes were not clinically significant.

The largest mean change in PR interval on C2D1 was a 2.8 ms (SD of 11.4) increase at 4 h postdose and a 6 ms (SD of 11.8) decrease at 24 h postdose. For categorical analysis, PR interval >200 ms but ≤220 ms was considered to be ACI, and PR interval >220 ms was considered to be APCS (CTCAE Version 4.0 guidelines). Three patients experienced at least one PR interval >200 ms with ECG status classified as ACI at those time points; of these, 2 had PR interval >200 ms at baseline and postdose. Two patients experienced at least one PR interval >220 ms with ECG status classified as APCS at their highest postdose PR reading time points. One patient had an average PR interval of 207 ms on C1D1 (1 h postdose), but the second ECG in the serial set of 3 ECGs had a PR reading of 235 ms, which was considered to be APCS.

The QRS duration was stable after AA administration. On C2D1, the largest mean increase of 1.0 ms (SD of 4.8) was observed at 0.5 h, and the largest decrease of −1.8 ms (SD of 4.2) was observed at 1.5 h. For categorical analysis, a QRS duration ≤100 ms was considered normal. Fourteen patients had QRS readings >100 ms at various time points during the study; of these, 13 had increased QRS duration during both baseline and postbaseline extractions. None of the patients had an increase in QRS duration from baseline by ≥10 % or an absolute value >120 ms (considered APCS).

Five patients had worsening of condition postdose based on ECG readings. Three patients with normal ECG predose experienced at least one instance of ACI classification in the ECGs postdose (sinus tachycardia or sinus bradycardia). Two patients with ECG readings classified ACI predose had at least one instance of APCS classification postdose (sinus bradycardia and first-degree AV block).

### Pharmacokinetic results

**Fig. 1:**
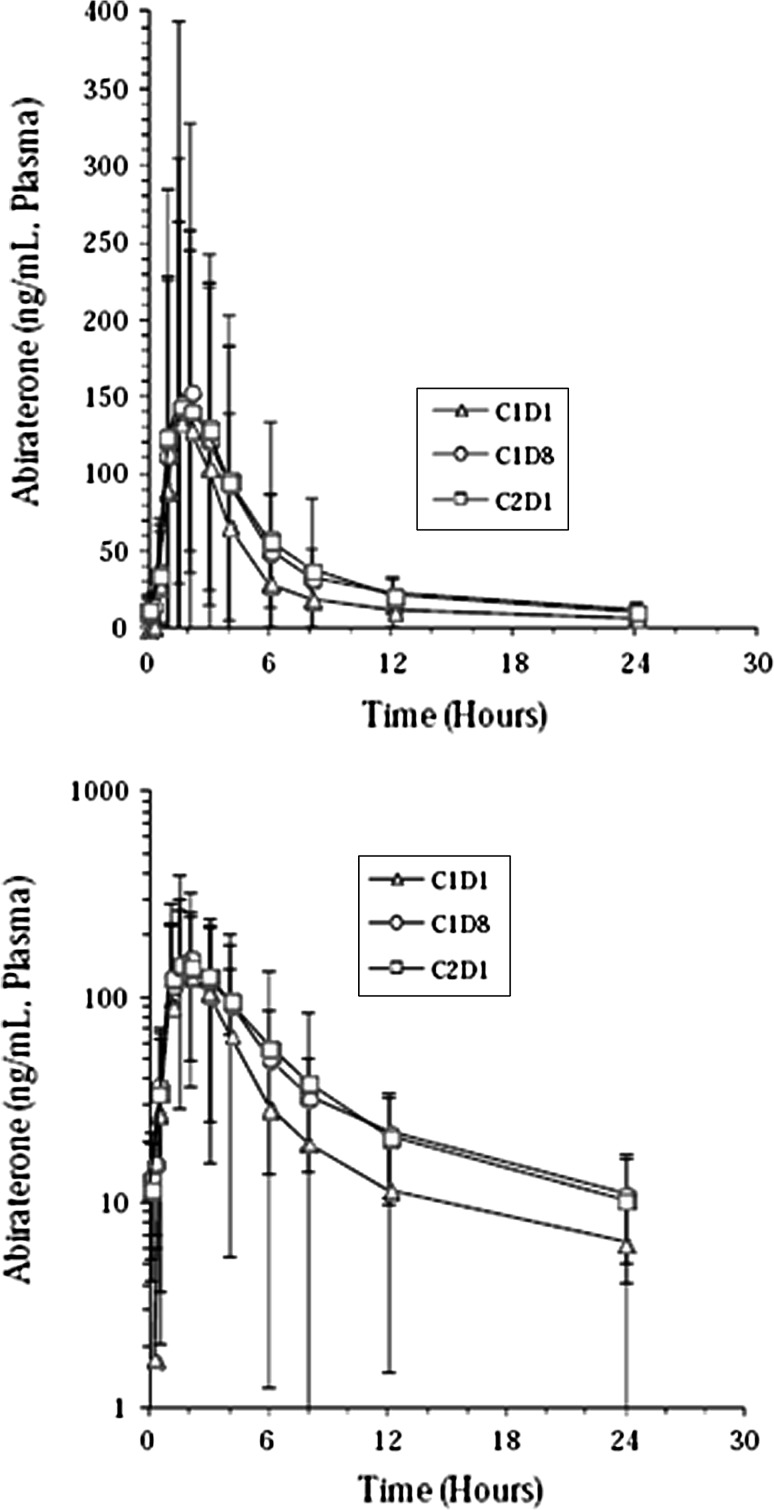
Mean (sd) plasma concentration–time profiles of abiraterone after single and multiple dose administration of abiraterone acetate to patients with metastatic CRPC. C1D1 = Cycle 1 Day 1; C1D8 = Cycle 1 Day 8; C2D1 = Cycle 2 Day 1; CRPC = castration-resistant prostate cancer

Since most AA concentrations in plasma were below the lower limit of quantification, PK analyses were not performed for AA. Peak abiraterone concentrations were reached at approximately the same time after single and multiple doses of AA (median, *t*_max_ ~ 2 h). Plasma abiraterone concentrations declined in a biphasic manner (Fig. 1). After a single dose of AA, systemic exposure was *C*_max_: 182 ng/mL and AUC_24h_: 675 ng h/mL. Systemic exposure values were comparable after multiple dosing on C1D8 and C2D1 (Table 3). Mean accumulation ratios were similar following multiple dosing (Table 3).

**Table 3:** Mean (SD) pharmacokinetic parameters following single-dose administration of abiraterone acetate (pharmacokinetic analysis set)

Parameter	Cycle 1 Day 1 (*N* = 33)	Cycle 1 Day 8 (*N* = 33)	Cycle 2 Day 1 (*N* = 33)
*C*_max_ (ng/mL)	182 (254)	207 (142)	226 (178)
*t*_max_ (h)	2 (1–4)	2 (1–4)	2 (1–6)
AUC_24h_ (ng h/mL)	675 (725)	965 (520)	993 (639)
AR *C*_max_	–	1.8 (1.8)	2.0 (2.4)
AR AUC	–	2.0 (1.5)	2.2 (2.3)

*AR* accumulation ratio, *AUC*_*24h*_ area under the plasma concentration–time curve from time 0 to 24 h postdose, *C*_*max*_ maximum plasma concentration

### Pharmacokinetic–pharmacodynamic relationship

**Fig. 2:**
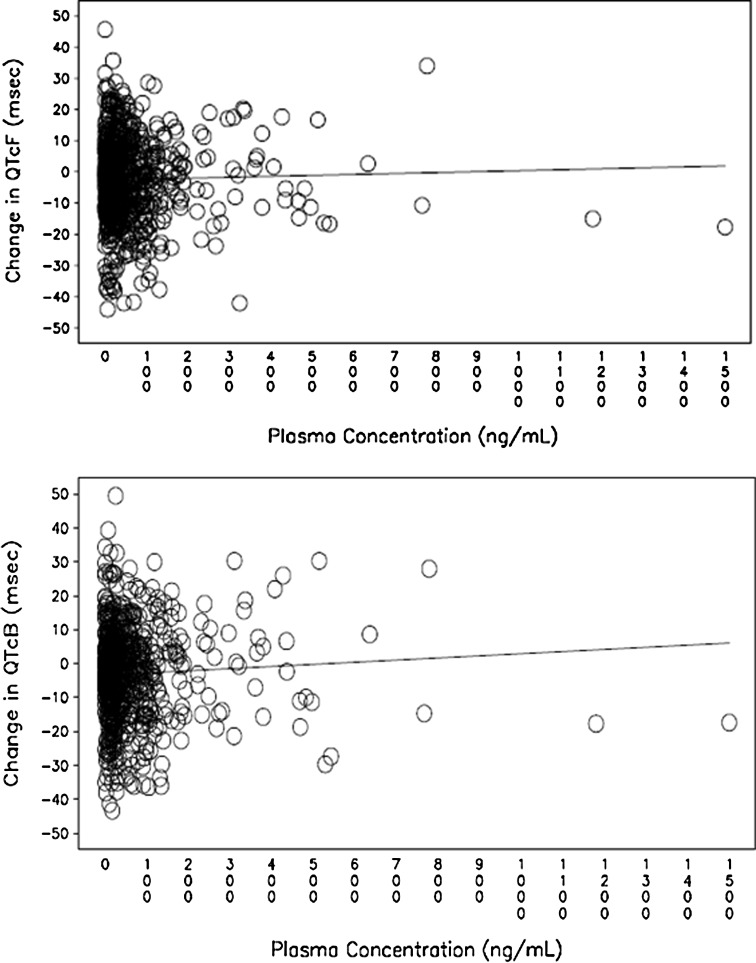
Scatter plot of plasma concentration of abiraterone versus change from baseline in QTcF and QTcB on day 1 of Cycles 1 and 2. The reference line for change in QTcF and QTcB was based on a linear mixed-effects model QTcF: intercept = −2.7015 (*P* = 0.0214) and slope = 0.0031 ms (*P* = 0.4737). QTcB: intercept = −3.4726 (*P* = 0.0124) and slope = 0.0064 ms (*P* = 0.1481)

The individual change from baseline in QTcF interval and corresponding abiraterone plasma concentrations exhibited no apparent relationship (Fig. 2). No significant correlations were observed between the change from baseline in QTcF and plasma concentration [estimated slope: 0.0031 ms; 90 % CI (−0.0040, 0.0102)] or between *C*_max_ and the corresponding change from baseline in QTcF at individual *T*_max_ [estimated slope: 0.0036 ms; 90 % CI (−0.0084, 0.0156)]. At mean C_max_ (204.1 ng/mL), the predicted values of mean change from baseline in QTcF and the associated 90 % CI [−1.99 ms (−5.2387, 1.2542)] further confirms the lack of correlation. Similar results were observed for QTcB (Fig. 2).

### Safety

Up to C2D2, 20 (61 %) patients reported at least one TEAE, all of Grade 1 or 2 (Table 4). A single Grade 3 TEAE of increased alkaline phosphatase (884 U/L; not serious, not drug-related, ongoing) was reported in 1 (3 %) patient. This patient had bone metastases at the time of enrollment, with baseline alkaline phosphatase of 378 U/L. The most commonly reported TEAE was hot flush (Table 4). Drug-related TEAEs were reported in 12 (36 %) patients, the most common were dizziness and hot flush in 4 (12 %) patients each. There were no Grade 4 TEAEs, serious TEAEs, TEAE leading to discontinuation, or deaths.

**Table 4:** Treatment-emergent adverse events in at least two patients (safety analysis set)

Preferred term	Total (*N* = 33)	Toxicity grade	
		1	2
Total patients with TEAEs	20 (60.6 %)	13 (39.4 %)	6 (18.2 %)
Hot flush	6 (18.2 %)	6 (18.2 %)	0 (0.0 %)
Dizziness	4 (12.1 %)	4 (12.1 %)	0 (0.0 %)
Peripheral sensory neuropathy	2 (6.1 %)	2 (6.1 %)	0 (0.0 %)
Back pain	3 (9.1 %)	0 (0.0 %)	3 (9.1 %)
Muscle spasms	3 (9.1 %)	3 (9.1 %)	0 (0.0 %)
Constipation	2 (6.1 %)	2 (6.1 %)	0 (0.0 %)
Nausea	2 (6.1 %)	2 (6.1 %)	0 (0.0 %)
Blood alkaline phosphatase increased	3 (9.1 %)^a^	1 (3.0 %)	1 (3.0 %)
Aspartate aminotransferase increased	2 (6.1 %)	2 (6.1 %)	0 (0.0 %)
Blood amylase increased	2 (6.1 %)	2 (6.1 %)	0 (0.0 %)
Fatigue	2 (6.1 %)	2 (6.1 %)	0 (0.0 %)
Hypokalemia	2 (6.1 %)	2 (6.1 %)	0 (0.0 %)
Dyspnea	2 (6.1 %)	2 (6.1 %)	0 (0.0 %)
Tumor pain	2 (6.1 %)	2 (6.1 %)	0 (0.0 %)

*TEAE* treatment-emergent adverse event

^a^The adverse event of blood alkaline phosphatase increased was of toxicity Grade 3 in one patient and was the only Grade 3 TEAE reported up to Cycle 2 Day 2; there were no Grade 4 toxicities reported

Five patients had TEAEs of interest: liver function test abnormalities [alkaline phosphatase increased (*n* = 2), aspartate aminotransferase increased (*n* = 1), both alkaline phosphatase and aspartate aminotransferase increased (*n* = 1)], hypokalemia (*n* = 2), and edema (*n* = 1). Most patients entered the study with Grade 0 or 1 hematology and chemistry values, and there were no shifts from Grade 0 or 1 to Grade 3, up to C2D2. Lymphocytopenia was reported as a Grade 2 abnormality in 3 patients. There were no reports of Grade 3 or 4 hematologic abnormalities and Grade 4 chemistry abnormalities.

## Discussion

Some of the non-cardiac drugs across diverse therapeutic classes have been known to delay cardiac repolarization, resulting in arrhythmias, especially concerning torsade de pointes [13]. Hence, the International Conference on Harmonization (ICH) E14 guidelines recommend rigorous characterization of the effect on the QT/QTc interval, for all new pharmaceutical agents, as part of their premarketing safety investigations [14]. For this study, a typical “Thorough QT/QTc Study” design, as per the ICH E14 guidelines, was not used for several reasons. First, a supratherapeutic dose could not be administered to patients because the safety and tolerability of doses exceeding 1,000 mg once daily has not been established. Secondly, a placebo arm was not possible due to the ethical issues associated with prolonged dosing of this population with a placebo.

This study was designed to conform with the “Intensive QT Trial” design, an alternative design to the typical “Thorough QT/QTc Study” design recommended in ICH E14 guidelines. This study is unique in its robust design compared to other Intensive QT Trials as it incorporated a full-day (over 24 h) time-matched baseline (predose) ECG time points on C1D-1 (instead of only one single predose baseline ECG time point) that allows for a comparison to time-matched ECGs postdose. Additionally, the patients were dosed to steady state, and ECGs were collected on C2D1 for analysis. This degree of rigor allows for an analysis of ECGs to identify drug accumulation effects, if they exist.

For QT correction, Fridericia’s formula was used, as it performs better with higher HRs than other formulae [15]. Additionally, as recommended in the ICH E14 guidance [14], data were also analyzed using Bazett’s formula with similar results (data not shown).

In this study, no clinically significant changes were seen in the QTcF with AA treatment. The upper bound of the two-sided 90 % CI for the baseline-adjusted mean change in QTcF duration across all postdose time points was below 10 ms, the suggested threshold of regulatory concern for non-antiarrhythmic drugs [14]. Neither absolute values nor changes from baseline in QTcF duration in this study are considered a clinical concern (i.e., no patient had a QTcF > 500 ms or a change in QTcF > 60 ms at any time point). Postdose QTcF increase >30 ms but <60 ms reported in two patients was not considered clinically significant.

Changes in HR, PR interval, and QRS duration were examined in this study, as these could reflect toxicities due to autonomic factors. However, the analysis of central tendency and categorical analysis of mean changes in these parameters did not show evidence of any clinically significant change on treatment with AA. Specifically, the lack of any clinically significant change in HR post-AA administration indicates that it is likely that both QTcF and QTcB data are not significantly affected by the changes in HR in this study. None of the patients had an increase in QRS duration from baseline by ≥10 % or an absolute value >120 ms.

Exposure increased after multiple dosing of AA compared with exposure after a single dose. The accumulation ratios following multiple dosing were similar. Steady-state concentrations appeared to have been reached after 1 week, as the accumulation ratios on C1D8 was similar to the accumulation ratios on C2D1. Maximum plasma concentrations were reached at a median *t*_max_ of 2 h. No relationship between change from baseline in QTcF and abiraterone plasma concentrations was observed. Similar results were observed for QTcB.

No deaths or serious TEAEs were reported through C2D2. All TEAEs were of Grade 1 or 2, except one Grade 3 TEAE of increased alkaline phosphatase. The toxicity grades of TEAEs observed are consistent with those reported in phase 2 and phase 3 studies with AA and prednisone [16, 17]. Up to C2D2, hypokalemia occurred in 2 patients, peripheral edema occurred in 1 patient, and there were no reports of hypertension. As the study population was entirely white, applicability of the study finding to other races is limited.

## Conclusions

There is no significant effect of AA plus prednisone on the cardiac QT/QTc interval using time-matched ECGs and PKs in patients with mCRPC. Systemic exposure to abiraterone increased upon multiple dosing. No relationship between change in QTcF and abiraterone plasma concentration was observed. The results indicate that AA does not affect the ventricular repolarization in humans to an extent that would impact risk–benefit considerations. AA plus prednisone treatment was generally well tolerated, and safety results were consistent with previous studies.
